# Correction of Dynamic Errors of a Gas Sensor Based on a Parametric Method and a Neural Network Technique

**DOI:** 10.3390/s16081267

**Published:** 2016-08-10

**Authors:** Jerzy Roj

**Affiliations:** Institute of Measurement Science, Electronics and Control, Silesian University of Technology, Gliwice 44-100, Poland; jerzy.roj@polsl.pl; Tel.: +48-32-237-1241; Fax: +48-32-237-2034

**Keywords:** gas sensor, dynamic correction, neural networks, response time, dynamic properties

## Abstract

The paper presents two methods of dynamic error correction applied to transducers used for the measurement of gas concentration. One of them is based on a parametric model of the transducer dynamics, and the second one uses the artificial neural network (ANN) technique. This article describes research of the dynamic properties of the gas concentration measuring transducer with a typical sensor based on tin dioxide. Its response time is about 8 min, which may be not acceptable in many applications. On the basis of these studies, a parametric model of the transducer dynamics and an adequate correction algorithm has been developed. The results obtained in the research of the transducer were also used for learning and testing ANN, which were implemented in the dynamic correction task. Despite the simplicity of the used models, both methods allowed a significant reduction of the transducer’s response time. For the algorithm based on the parametric model the response time was shorter by approximately eight-fold (reduced up to 40–80 s, i.e., about 2–4 sample periods), whereas with the use of an ANN the output signal was practically fixed after a time equal to one sampling period, i.e., 20 s. In addition, the use of ANN has allowed reducing the impact of the transducer dynamic non-linearity on the correction effectiveness.

## 1. Introduction

Various types of gas sensors have quite a long response time to a step change in gas concentration. At the beginning there is a transient state, which is dominated by the phenomenon of adsorption (or desorption) of gas molecules in the sensitive layer of sensor. At the end the equilibrium is established and the output signal of the transducer reaches a steady value. This transient state can take quite a long time, from a few, to several, minutes. This is due to two reasons: the phenomenon of chemical adsorption in a sensitive layer occurs relatively slowly [1,2,3,4,5,6,7], and often additional filters and screens are used for example for separation of undesirable substances. In many measurement applications such a long time may be unacceptable for industrial and safety systems. The possibilities of shortening the response time by a suitable design of the sensor or applying better materials of the sensing layer are still being examined [8,9,10]. In some cases, measurements are based on the specific use of transient characteristics of the sensor [11,12,13,14].

Shortening the response time of the transducer is also possible by the use of a correction procedure implemented by various methods in the next section of the measuring chain. Technical realization of such a correction may be relatively simple and does not necessarily entail the expansion of the measuring chain, because due to the static properties of the sensors (non-linearity, non-selectivity) a procedure of correction of these features is necessary in any case. The basic structure of a transducer to measure gas concentration is shown in Figure 1.

Changes in gas concentration *C* in the atmosphere surrounding the sensor, due to the phenomenon of adsorption of gas molecules, causes changes of the sensitive layer properties. Consequently, adequate changes of the electrical properties (resistance, permittivity) occur. The sensor can be conventionally divided into two parts. The dynamic part corresponds to a slow adsorption (or desorption) of particles on the surface of the sensitive layer including the possible permeation through the filter, if used. The static part represents the changes of the electrical parameter of the layer as a result of molecule adsorption. Finally, the conditioning circuit produces an output signal *u*, which depends on the gas concentration. This circuit is treated as a static one. Supplementing the measuring chain by the algorithm of dynamic correction allows the corrected output signal *u*_c_, whose value is fixed after a shorter time, to be obtained. That is the issue discussed in this article in relation to two methods of dynamic correction. The first method is based on knowledge of the structure and parameters of the dynamic model of the sensor and the second one involves using an ANN. Research was carried out for a gas concentration-measuring transducer described in [15] with a solid-state resistive sensor with a SnO_2_ sensitive layer (type TGS2442 Figaro, Glenview, IL, USA).

The main motivation of this article is to propose two relatively simple methods to shorten the response time of the low-cost semiconductor gas sensors, which typically have poor dynamic properties.

## 2. Investigation of the Sensor Dynamics

The analysis of gas sensor dynamic properties carried out in a theoretical manner or by a simulation method [1,2,3] has a significant cognitive meaning. It can be the basis for obtaining a general form (structure) of the dynamic model and, consequently, the dynamic correction algorithm. However, in practice, it is necessary to introduce some measurement results to obtain numerical values of parameters of such a model. In the case of gas sensors it is relatively easy to perform the step response method. Figure 2 shows a scheme of the used measurement system.

Abrupt changes of the gas concentration are obtained by rapid changes of the flow in the channel of the respective constituent gases. Mixing of gases takes place in the inlet channel, so that the concentration in the measuring chamber is already fixed. It is essential that the rise time is sufficiently short. This creates the need for small dimensions of both the inlet channel and the measuring chamber and, at the same time, the use of a high flow rate of the gas mixture. These parameters were chosen in such a way that the rise time of the gas concentration was less than 10 s.

During the tests the gas concentration was changed abruptly as shown in Figure 3a. The resulting changes in the output signal are shown in Figure 3b.

Analysis of the waveforms indicates the inertial nature of the sensor—the output voltage *u* grows slowly until a steady-state value. Simple, but suitable in this case, are the dynamic models of the first or second order, which may be the basis for the development of the dynamic correction algorithm in the time domain, which can be expressed by the following equations:

(1) $$T\;\frac{du(t)}{dt}+u(t)=kC(t-T_{0})$$

(2) $$T_{1}T_{2}\;\frac{d^{2}u(t)}{dt^{2}}+\left(T_{1}+T_{2}\right)\;\frac{du(t)}{dt})+u(t)=kC(t)$$

where *C* and *u* are measured gas concentration and output voltage, respectively (see Figure 1), *t* is time, *T*, *T*_1_, *T*_2_ (time constants), and *T*_0_ (delay time) are parameters related to the sensor dynamics, and the coefficient *k* (sensitivity) expresses its static properties. In the presented considerations, determining the sensitivity is not necessary because the subject of the correction are only the dynamic properties and the correction relates to the output voltage of the converter. Problems concerning the correction of the static characteristics of the transducer are discussed in more detail in [16].

As is visible on Figure 3b the constant value of the output signal corresponding to the actual constant value of gas concentration is obtained after more than ten minutes. A dynamic error may be defined as *e*(*t*) = *u*(*t*) − *kC*. This error is a function of time and decreases to zero for a steady state. The time interval *t*_r_ from a step change in gas concentration until the error *e*(*t*_r_) is less than 5% of the measuring range, and is called the response time of the measuring transducer.

## 3. Correction Based on the Dynamic Parametric Model of Sensor (Method 1)

### 3.1. Identification of the Dynamic Model

In the traditional approach to the development of an algorithm of dynamic correction the structure of the model and values of its parameters must be known. This data can be obtained by the analysis of results of the experiment described above. In spite of the fact that the simplest model of the first order given by the Equation (1) can be useful, the second order model (Equation (2)) was also taken into account for comparison. For the convenience of further consideration these models are expressed by the corresponding equations in the *s*-domain (using Laplace transform) as the following transfer functions:

(3) $$K_{\text{t1}}(s)=\frac{k}{sT+1}e^{-T_{0}s}\text{ ; }K_{\text{t2}}(s)=\frac{k}{\left(sT_{1}+1\right)\left(sT_{2}+1\right)}$$

where parameters of the model *T*, *T*_1_, *T*_2_ (time constants), and *T*_0_ (delay time) are determined on the basis of the measurement data (Figure 3b).

For all phases of the waveform shown in Figure 3b each step response was separated and each one was scaled relative to the steady state values having regard to the direction of the concentration change. The selected set of step responses, including those for the largest and smallest values of the time constants, is shown in Figure 4.

It is evident that the time constants do not have the same values for the concentration changes of various ranges. This means the sensors are dynamically non-linear. The proper implementation of dynamic correction would require taking into account the changes in the dynamic model parameters. However, this study assumed the use of a simplified linear model with the time constant calculated as an average value of the individual time constants for all of the phases. The obtained results of dynamic correction, discussed in Section 3.3, shows that this simplification leads to small dynamic errors. They can be omitted in a first approximation in comparison with errors arising from the imperfect static non-linearity correction and correction of influencing quantities [16]. The averaged time constants of the dynamic models of the sensors were determined based on the average step response—the obtained values are given in Table 1.

### 3.2. Algorithm of the Dynamic Correction

A block diagram shown in Figure 5 illustrates the principle of the proposed dynamic correction method. The dynamic model of the transducer composed of the sensor and a signal conditioner is expressed by the transfer function *K*_s_(*s*), whereas *K*_t_(*s*) is a transfer function for the whole system (transducer supplemented with dynamic corrector). For both of them, the same type of model is assumed. In order to shorten the response time by a factor α = (0,…,1), the time constant for the whole transducer can be expressed as α*T*, so the transfer function of a dynamic corrector can be derived as:

(4) $$K_{c}(s)=\frac{U_{c}(s)}{U(s)}=\frac{U_{c}(s)}{C(s)}\frac{C(s)}{U(s)}=K_{t}(s)K_{s}^{\text{-1}}(s)=\frac{k}{sT\alpha +1}{\left(\frac{k}{sT+1}\right)}^{-1}=\frac{sT+1}{sT\alpha +1}$$

where *U* and *U*_c_ denote the values of the voltage before and after dynamic correction (see Figure 1). The same result is obtained when both models (1) and (2) are considered and the reduction concerns the dominant (the greater) time constant. It means that it is *T*_0_ << *T*_1_ or *T*_2_ << *T*_1_ in case (1) or (2), respectively.

After sampling the signal (which is natural in respect of the numerical dynamic correction algorithm) and using the Tustin method to convert models from continuous time into discrete time, a recurrent algorithm of the dynamic corrector can be derived from Equation (4). It has the following form:

(5) $$u_{c}(n)=\frac{2T+T_{s}}{2\alpha T+T_{s}}u(n)-\frac{2T-T_{s}}{2\alpha T+T_{s}}u(n-1)+\frac{2\alpha T-T_{s}}{2\alpha T+T_{s}}u_{c}(n-1)$$

where *T*_s_ is the constant sampling period, *u*(*n*) and *u*(*n* − 1)—output voltage before dynamic correction while *u*_c_(*n*) and *u*_c_(*n* − 1) are these after correction in actual *n* and previous *n* − 1 cycles of the recurrent calculation.

### 3.3. Efficiency of Correction

Figure 6 shows an example of application of the dynamic correction algorithm to reconstruct the gas concentration variation in time. The transducer mentioned in the first part was tested. Abrupt changes in concentration followed every 10 min. The parameter α was set equal to 1/4, which means a four-fold reduction of the response time is postulated. This parameter can theoretically be arbitrarily small, but in practice there is no sense to set α less than the quotient of the applicable sampling time *T*_s_ and the dominant time constant of the sensor *T*_1_ (in this case approx. 20/120 = 1/6).

The waveform presented in Figure 6 confirms the effectiveness of the above-described dynamic correction method. The response time without correction is eight to ten minutes and after applying the correction in most cases it does not exceed one minute. There are visible small overshoots, mostly for low gas concentrations. This is due to non-ideal matching of the averaged model—the average time constant used in the calculation is not well matched for all ranges of gas concentration. Due to the overshoot effect the algorithm was modified—if the calculated output voltage *u*_c_ has negative value, it is set to zero (the last phase in the sequence in Figure 6).

## 4. Correction Based on an Artificial Neural Network (Method 2)

ANNs are widely used in measurement techniques, which is a consequence of their basic features: the ability to learn and generalize, that is, the ability to generalize the acquired knowledge for new, previously-unknown data. This enables avoiding the necessity of complete and sufficiently precise formal modelling of the analog processing chain, the operation of which can be relatively easily described by “examples”. Especially good approximation properties have feed-forward multilayer neural networks with sigmoidal transfer functions, called Multi Layer Perceptron (MLP) networks or Back Propagation (BP)-type networks [17,18]. These networks are also often used in devices with semiconducting metal oxide-based sensors for detection and concentration estimation of various gases. In many cases, sufficiently good results are obtained with MLP networks with one hidden layer [19,20,21]. In more complex cases generally are sufficient MLP networks with two hidden layers [22,23].

### 4.1. Real-Time Dynamic Error Correction Algorithm

In general, the dynamic properties of analog transducers are usually modelled by a linear differential equation of the *n*-th order [24,25,26]. The real-time dynamic correction algorithm can be achieved by presenting this equation as a system of *n* first order equations in a discrete state space [27,28] and then solving this system due to the input quantity. A detailed description of this algorithm and its neural network implementation is given in [29].

The main disadvantage of the “classic” dynamic correction algorithms is necessary to identify the coefficients of the differential equation describing the dynamics of the sensor. It is required to determine the coefficients of the algorithm which, in general, is a linear combination of these coefficients and subsequent measurement results (e.g.,—Section 3.2).

In the simplest case, the dynamic model of the transducer can be described by a linear first order differential Equation (1) which for *T*_0_ = 0 has the form:

(6) $$T\frac{du(t)}{dt}+u(t)=kC(t)$$

The real-time dynamic correction algorithm takes the form [29,30,31]:

(7) $$u_{c}(n)=\frac{1}{\psi }\left[u(n)-\phi \;u(n-1)\right]$$

in which *u*_c_(*n*) is the corrected value of the *u*(*t*_n_) voltage (Figure 1) at the *t*_n_ moment of time, (*n* = 0, 1, …, *N*,…) and:

(8) $$\phi =\exp\left(-\frac{T_{s}}{T}\right),\;\psi =1-\exp\left(-\frac{T_{s}}{T}\right)=1-\phi$$

where *T*_s_ is the sampling period, and *T* is the time constant.

As can be seen, the designation of the coefficients ϕ and ψ requires the identification of the dynamic model time constant *T* which, in turn, requires a separate process of the model identification. It can be difficult to implement, ineffective, time consuming, and costly, especially for the higher order dynamic model of the sensor. An alternative in such situations is to use an ANN [17,18,32] which “performs” this type of identification in the learning process.

### 4.2. Neural Network Implementation of the Real-Time Dynamic Error Correction

Presenting Equation (7) in the form:

(9) $$u_{c}(n)=w_{1}u(n-1)+w_{2}u(n)$$

where $w_{1}=-\frac{\phi }{\psi }$ and $w_{2}=\frac{1}{\psi }$, processing of the linear neuron shown in Figure 7a (*u_c_*(*n*) = *z* for linear transfer function) is obtained. In the ideal case, when the sensor is described by a linear first order differential Equation (6) and the measurement results are free of errors, the result of dynamic correction is achieved after a time equal to the sampling period *T*_s_. It can be concluded that the linear neuron during the learning process performs indirect identification of the time constant *T*.

For the practical verification of the dynamic correction effectiveness implemented by a single linear neuron, the learning process was carried out. This process uses the same dataset as for identification of the dynamic model of the sensor described in Section 3.1. The results of the dynamic correction for the test data are presented in Section 4.3.

In order to take account of the dynamic non-linearity of the sensor, the neural network was expanded by a hidden layer with the sigmoidal [17,18] transfer functions (became an MLP-type neural network). During the research, the ANNs were tested with the number of hidden neurons from 2 to 16, which has shown that in a given case four neurons in the hidden layer are sufficient, so the ANN has the 2-4-1 structure illustrated in Figure 7b. Increasing the number of neurons in the hidden layer or increasing the number of layers did not result in a significant correction improvement.

According to Figure 7b, processing equations of the ANN are as follows:

(10) $$s_{i}=w_{1i}\;u(n)+w_{2i}\;u(n-1)+b_{i}\;for\;i=1,\;...4$$

(11) $$h_{\;i}=\frac{1}{1+\exp(-s_{i})}\;for\;i=1,\;...4$$

and:

(12) $$u_{c}(n)=z=\sum_{j=1}^{j=4}v_{j}h_{j}+b_{0}$$

where *w_ji_*, *v_j_* are weights, and *b_j_*, *b*_0_ are biases of the individual neurons.

### 4.3. Efficiency of the Correction Performed by ANN

Figure 8a shows the results of dynamic correction implemented by a single linear neuron (Figure 7a). There are correction errors resulting from the assumption of the first order linear model to describe the dynamics of the sensor and random errors resulting from the quantization process additionally increased by the correction algorithm. Dynamic correction errors resulting from the quantization process can be reduced by increasing the resolution of an A/D converter [29] and/or by increasing the sampling period *T*_s_ [33].

In turn, Figure 8b illustrates the results of the dynamic correction implemented by the ANN with a 2-4-1 structure (Figure 7b). It can be seen that the addition of a hidden layer improved the dynamic correction quality compared to a single linear neuron. However, the effect of the random error amplification can still be seen. In the cases where errors will exceed the limit values, a variety of filtering or smoothing algorithms can be used [34,35,36].

## 5. Conclusions

Despite the simplicity of the applied dynamic models for the gas sensor, the dynamic correction algorithms derived from them are very effective—the transducer response time was significantly reduced.

For a more detailed evaluation of the effectiveness of the proposed dynamic correction methods, an dynamic error *e*(*t*) has been defined as the difference between the voltage value obtained as a result of the correction *u*_c_(*t*) and the voltage value in a steady state, which corresponds to the measured gas concentration. Such calculated error values for the two described methods are shown in Figure 9. These methods were compared taking into account three main aspects: the degree of shortening the response time, the impact of the sensor dynamic non-linearity and the impact of random errors.

The transducer response time without correction is about 7–8 min. Both methods used for the dynamic correction allow shortening this time significantly. In the case of Method 1, the response time does not exceed 60 s, whereas in the case of Method 2 (ANN), the time is equal to the sampling period (in the conducted studies: 20 s). The exception is the case of a very small concentration (curve labeled “e1” in Figure 9), for which the response time is twice as long in both methods.

Method 1 does not take into account the dynamic non-linearity of the sensor due to the use of the average model. In this case the degree of shortening the response time depends on the range of changes of the gas concentration and varies between 40 and 90 s. However, in Method 2 the dynamic non-linearity of the sensor can be (to some degree) taken into account by extending the network of the hidden layer with non-linear transfer functions. The research showed that a simple 2-4-1 ANN structure allows obtaining a measurement result after time equal to the sampling period, regardless of changes in the concentration range.

However, it should be noted that the ANN-based dynamic error correction is more sensitive to the random errors of input data than the algorithm proposed in Section 3 (Figure 9a vs. Figure 9b). In a situation when the corrected results contain random errors exceeding limit values several actions can be taken to reduce them. The simplest way is to increase the resolution of the A/D converter. There may also be different smoothing algorithms used or the sampling period may be increased. However, in both cases this leads to an increase in the time to obtain the measurement result.

The application of an ANN technique to the dynamic correction has some significant advantages. The most important of them is that it is not necessary to identify neither the dynamic model of the sensor nor calculate the correction algorithm coefficients. This task is performed during the learning process of the neural network. In addition, a very simple structure of the ANN is sufficient for effective correction of the dynamic errors of a gas transducer.

## Figures and Tables

**Figure 1:**
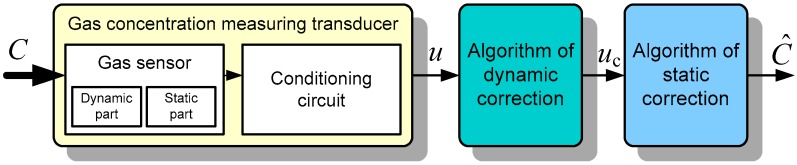
Structure of the gas concentration transducer with dynamic and static correction.

**Figure 2:**
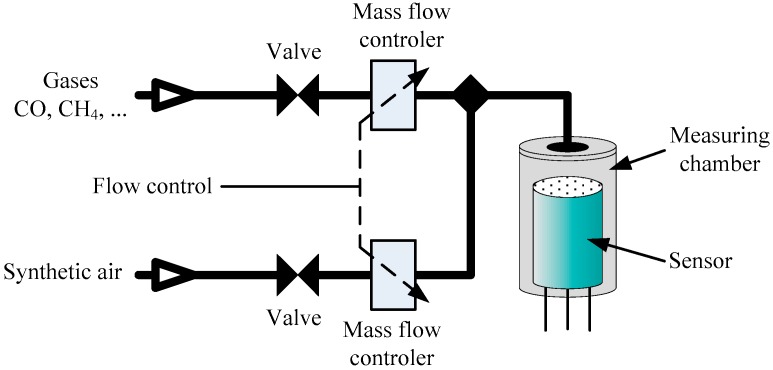
Simplified scheme for gas sensor investigations.

**Figure 3:**
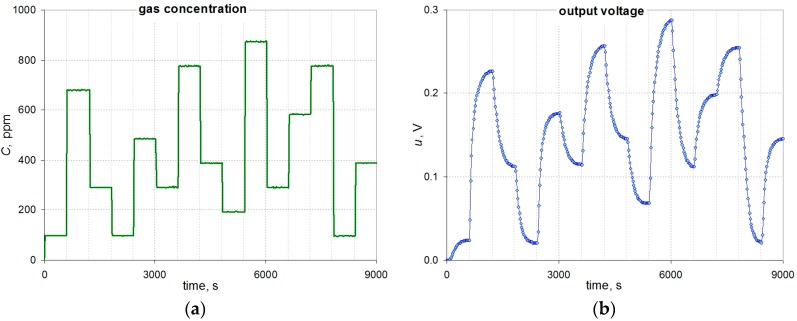
Changes in the gas concentration (**a**) and the corresponding changes of the output voltage (**b**).

**Figure 4:**
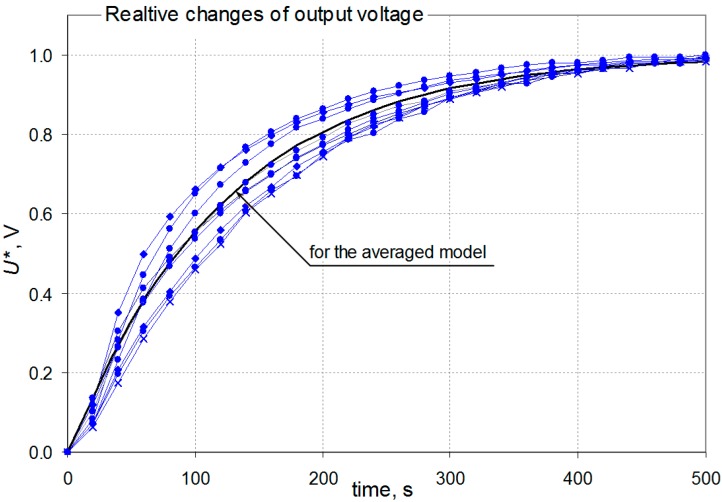
Scaled step responses for selected phases of the sequence shown in Figure 3b.

**Figure 5:**
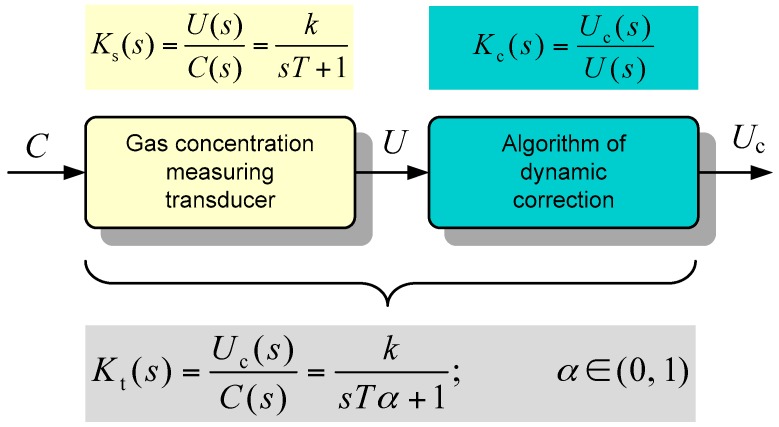
Transfer functions expresses dynamic models of all parts of gas concentration measuring transducer (*k* represents the sensitivity of sensor in steady-state).

**Figure 6:**
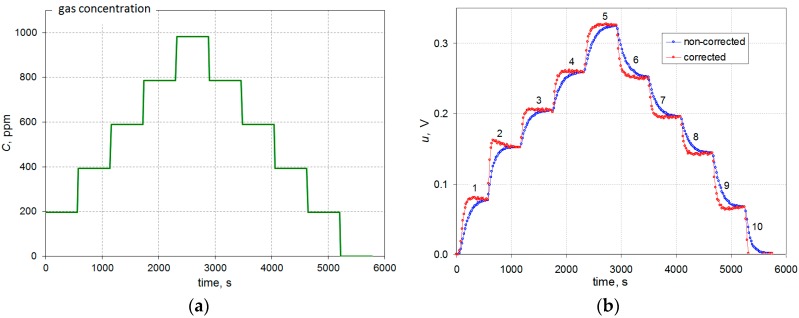
Illustration of the effectiveness of dynamic correction algorithm used for the test sequence; changes of gas concentration (**a**) and responses of the transducer (**b**).

**Figure 7:**
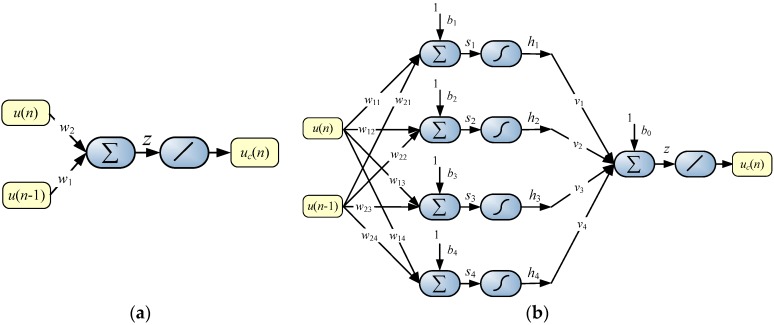
Structures of ANNs realizing real-time dynamic error correction (**a**) linear neuron in the case of the sensor model described by first order linear differential Equation (7); (**b**) 2-4-1 structure of the ANN which allows taking into account the non-linearity of the sensor’s dynamic.

**Figure 8:**
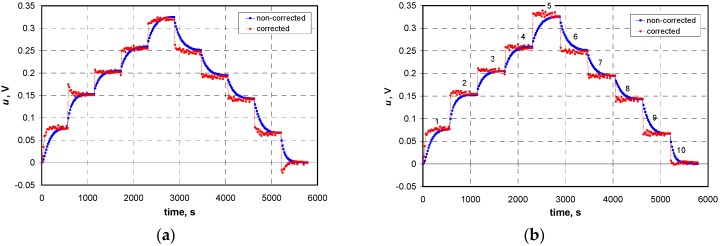
Illustration of the effectiveness of the real-time dynamic correction used for test data and performed by (**a**) linear neuron (Figure 7a); and (**b**) the ANN from Figure 7b.

**Figure 9:**
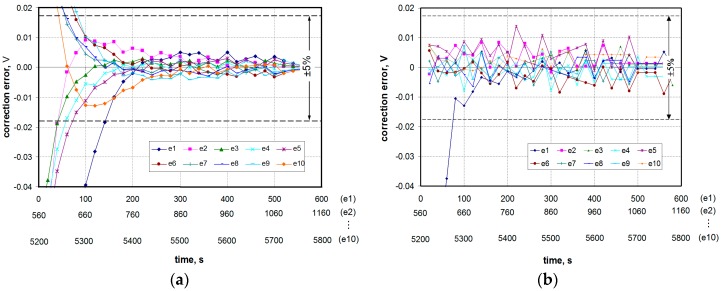
Errors of dynamic correction carried out by the algorithm described in Section 3.2 (**a**) and ANN having the 2-4-1 structure (**b**). Symbols e1...e10 refer to numbered fragments of sequences in Figure 6b and Figure 8b.

**Table 1:** A set of parameters of the dynamic models.

Type of Model	Averaged Parameters	Maximum	Minimum
First-order	*T* = 126.5 s	144.6 s	95.7 s
with delay	*t*_0_ = 21.2 s	43.2 s	0.0 s
Second-order	*T*_1_ = 126.5 s	144.6 s	95.7 s
	*T*_2_ = 24.4 s	40.6 s	0.0 s
